# Frequency of *GNAS* R201H substitution mutation in polyostotic fibrous dysplasia: Pyrosequencing analysis in tissue samples with or without decalcification

**DOI:** 10.1038/s41598-017-03093-1

**Published:** 2017-06-06

**Authors:** Su-Jin Shin, Seok Joo Lee, Sang Kyum Kim

**Affiliations:** 1 Department of Pathology, Hanyang University College of Medicine, Seoul, Korea; 2 Department of Pathology, Yonsei University College of Medicine, Seoul, Korea

## Abstract

Guanine nucleotide-binding protein/α-subunit (*GNAS*) mutations are involved in fibrous dysplasia (FD) pathogenesis. Here, we analyzed *GNAS* mutations in FD which were performed by pyrosequencing DNA isolated from formalin-fixed paraffin-embedded (FFPE) tissue. The mutation detection rate was determined in FD specimens with and without decalcification. *GNAS* mutation was identified in 28 cases out of 87 FDs (32.18%) [p.R201C (*N* = 14) and p.R201H (*N* = 14)]. *GNAS* mutation was more likely to occur in polyostotic FD (7/28, 25.0%); FD without *GNAS* mutation was mostly monostotic form (56/59, 94.9%, *P* = 0.011). The G > A (R201H) mutation was more frequent in polyostotic FD (6/14 patients, 42.9%) than the C > T (R201C) mutation (1/14, 7.1%) (*P* = 0.077). We divided the FD cases into two subgroups: tissue specimens that were not decalcified (*N* = 35, 40.2%), and tissue specimens that were decalcified (*N* = 52, 59.8%). *GNAS* mutation was more frequently identified in FD specimens that were not subjected to decalcification (23/35, 65.7%) than in FD specimens that were decalcified (5/52, 9.6%) (*P* = 0.001). In conclusion, mutation analysis of *GNAS* by pyrosequencing has diagnostic value in FFPE tissue of patients with FD, especially in specimens that were not decalcified. The R201H substitution mutation of *GNAS* may be involved in the pathogenesis of polyostotic FD.

## Introduction

Fibrous dysplasia (FD) is a frequently encountered benign fibro-osseous lesion. FD is histologically characterized by fibrous tissue with bland-looking fibroblasts, irregularly shaped woven bone, and no osteoblastic rimming. FD can be involved in one site (monostotic disease) or several sites (polyostotic disease) of any bone. In approximately 5% of all cases, polyostotic disease may be associated with McCune-Albright syndrome^1^. Monostotic and polyostotic FD, McCune-Albright syndrome, and soft tissue myxoma with FD all result from somatic mutation of the same guanine nucleotide-binding protein/a-subunit (*GNAS*) gene^2–4^.

*GNAS* is located on chromosome 20q13.3, which encodes the α-subunit of the heterotrimeric G (Gsα) protein complex^4–6^. *GNAS* mutations induce the activation of G-protein a-subunit and cause FD, also known as McCune-Albright syndrome^5–7^. FD is thought to be predominantly caused by two mutations in exon 8 of the GNAS gene^8^. These mutations are substitutions in codon 201, which lead to the replacement of arginine 201 with histidine (R201H) or cysteine (R201C). Substitution of arginine 201 with serine (R201S)^9^, leucine (R201L)^10, 11^, and glycine (R201G) have been reported in rare cases^12^. One study reported a point mutation at codon 227 (glutamine) in exon 9 of *GNAS*, which led to replacement with leucine (Q227L)^13^.

Several studies showed that no *GNAS* mutations were detected in all other benign fibro-osseous lesions, and suggested that mutation analysis of *GNAS* is a reliable and valuable diagnostic tool for FD. A meta-analysis of 203 patients with FD reported the positive detection of *GNAS* mutation in 71.9% of the patients^14^. However, the detection rate in subsequent studies varied from 45–95% due to tissue mosaicism and sensitivity of the technique^15–19^.

Several methods can be used to detect *GNAS* mutations in paraffin-embedded tissues or frozen samples, including Sanger direct sequencing^14, 17^, allele-specific PCR^20^, PCR with mutation-specific restriction enzyme digestion^13^, and coamplification at lower denaturation temperature PCR (COLD-PCR)^4^. Recently, Liang *et al*. reported a detection sensitivity of *GNAS* mutation as high as 95% using next-generation pyrosequencing^19^. They isolated DNA from undercalcified, formalin-fixed, methylmethacrylate-embedded tissue. However, most other studies used tissue specimens that had been subjected to various decalcification methods before preparing formalin-fixed paraffin-embedded (FFPE) FD tissue samples. Calcified tissues were treated with strong acid for decalcification before fixation and embedding. This harsh treatment may affect DNA integrity, and reduce the quantity and quality of DNA for amplification and sequencing^21^.

In this study, we evaluated the effect of tissue decalcification on the diagnosis of FD by pyrosequencing for *GNAS* mutations. We selected FFPE specimens of patients with FD according to whether they had undergone decalcification or not, and used these samples to detect *GNAS* mutations. We also analyzed clinicopathological features according to *GNAS* mutation status.

## Results

### Clinical and pathological features

The clinical features of 87 patients with FD are presented in Supplementary Table 1. The male-to-female ratio of patients with FD was approximately 1:1 (43 males and 44 females). The median onset age was 31.02 years (range, 1–85 years). A total of 34 patients had FD located at craniofacial sites (34/87, 39.1%), and the remainder were located at extracraniofacial sites (53/87, 60.9%). Ten patients had multiple bone lesions at FD diagnosis, which radiologically suggested polyostotic FD. One of these 10 patients was diagnosed with Albright-McCune syndrome. Tissue samples were not decalcified before formalin fixation for 35 patients; the tissues samples of the remaining 52 patients were decalcified before formalin fixation.

**Figure 1:**
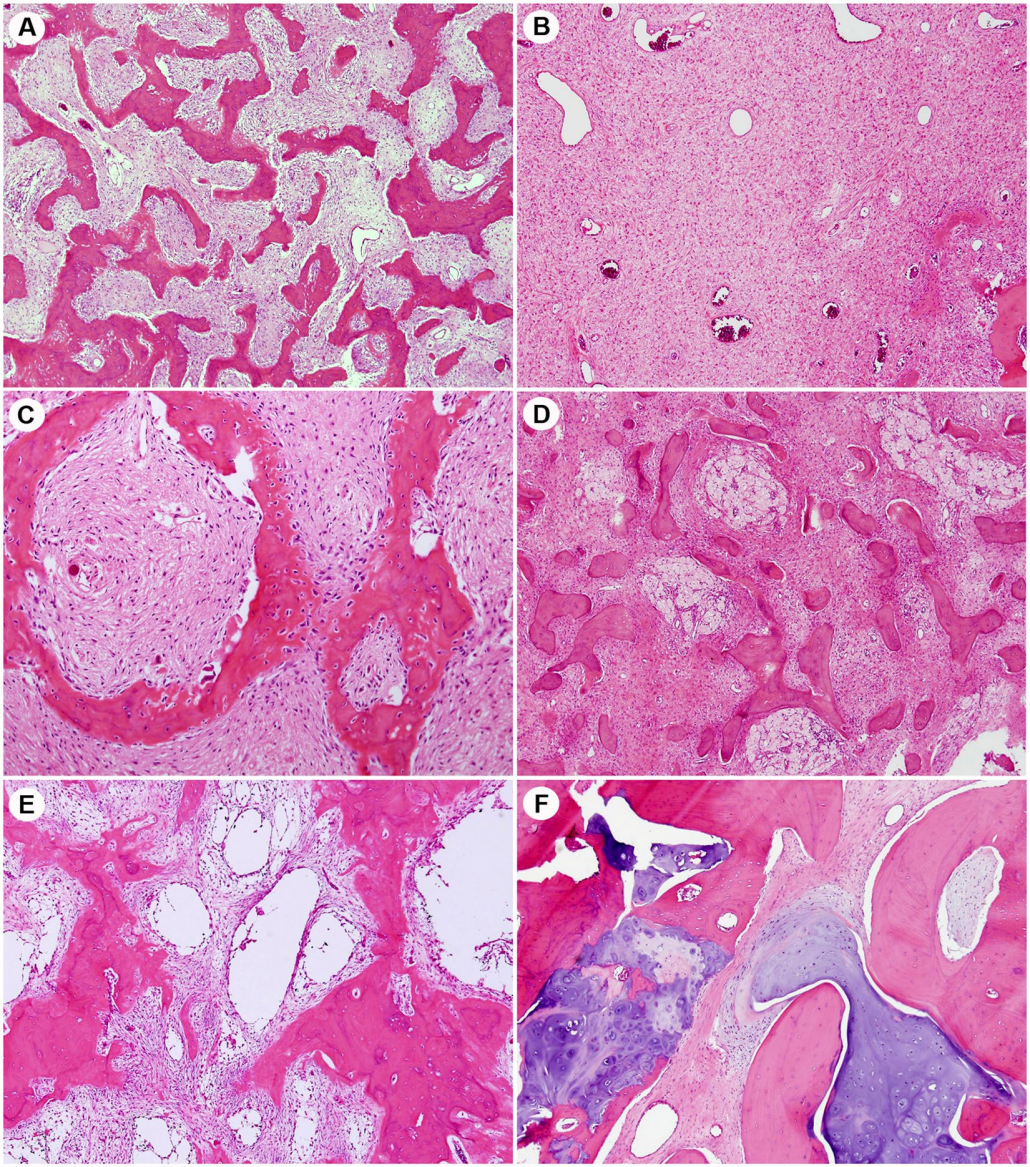
Histological features of fibrous dysplasia, which contains fibrous tissue with irregular, curvilinear, and trabeculae of woven bone in varying proportions. (**A**) Low fibrous tissue. (**B**) Abundant fibrous tissue. (**C**) Typical irregularly shape woven bones without osteoblastic rimming were observed in most cases. Some cases showed (**D**) aggregation of foamy histiocytes and (**E**) cystic changes. (**F**) Benign cartilaginous differentiation was observed in one case.

Histological examination indicated that most FD lesions contained varying proportions of fibrous tissue with irregular, curvilinear, and trabeculae of woven bone without osteoblastic rimming (Fig. 1). Some samples displayed aggregation of foamy histiocytes and cystic change. Benign cartilaginous differentiation was observed in one sample.

### *GNAS* mutation in fibrous dysplasia

**Figure 2:**
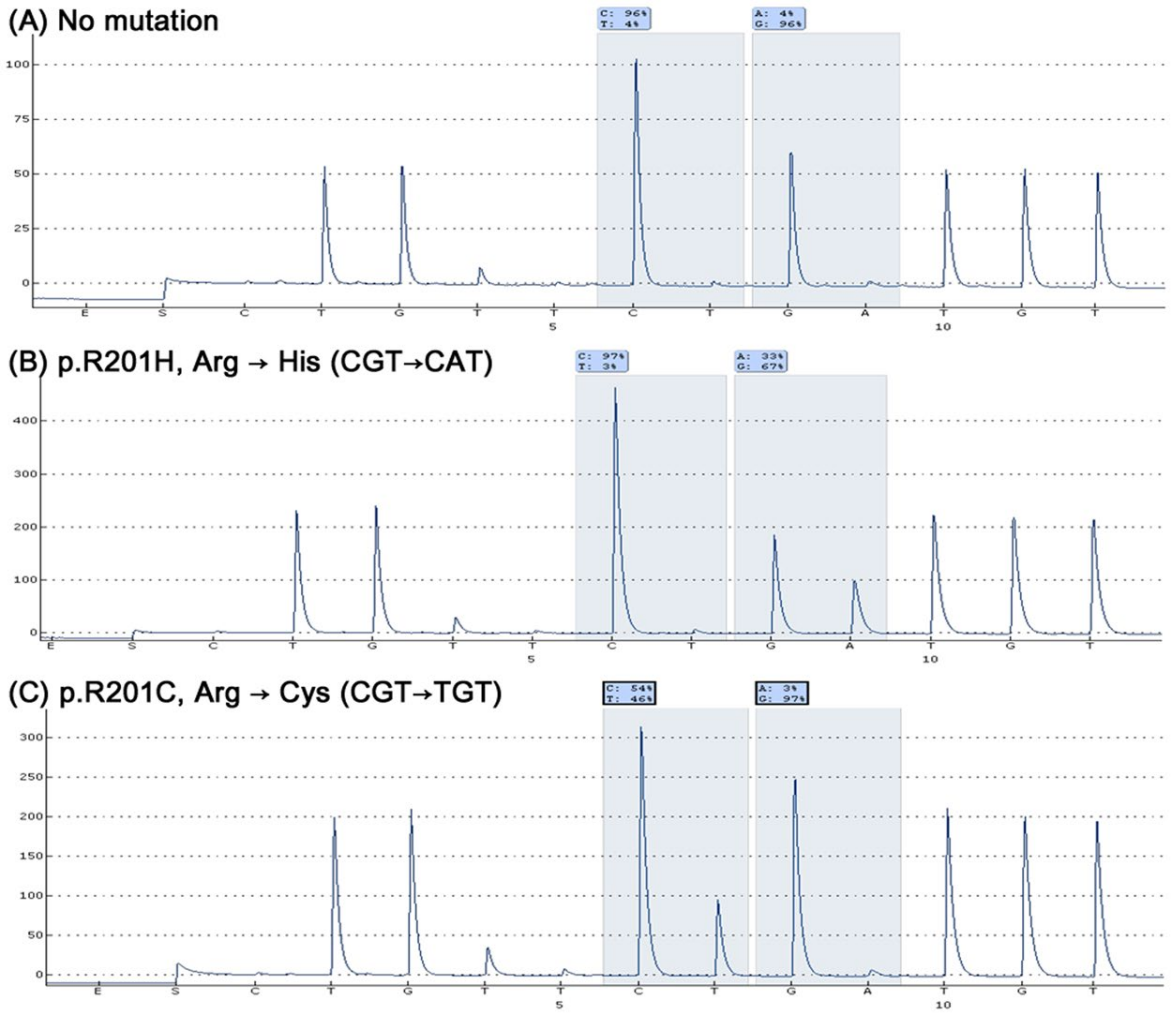
Typical pyrogram of (**A**) wild-type, (**B**) R201H (CGT → CAT), and (**C**) R201C (CGT → TGT) mutations of *GNAS* in fibrous dysplasia.

We performed pyrosequencing and detected *GNAS* mutations in 28 out of 87 patients with FD (32.2%, Table 1). Fourteen cases had the R201H mutation, and 14 cases had the R201C mutation (Fig. 2). We divided the FD patients into two groups, FD with *GNAS* mutation and FD without *GNAS* mutation, and compared clinical features in the two groups (Table 1). The results showed that polyostotic disease was observed more frequently in FD cases with *GNAS* mutation (7/28, 25.0%) than in FD without *GNAS* mutation (3/59, 5.1%, *P* = 0.011). However, there were no significant differences between the two groups with respect to gender, age at diagnosis, or lesion site.

**Table 1:** Clinical findings in fibrous dysplasia patients according to *GNAS* mutations status and cysteine or histidine substitution.

	*GNAS* mutations status			Cysteine or histidine substitution		
	Absence of mutation (n = 59, 67.8%)	Presence of mutation (n = 28, 32.2%)	*P*-value	p.R201C (n = 14)	p.R201H (n = 14)	*P*-value
Gender			1.000			0.706
Female	30 (50.8%)	14 (50.0%)		6 (42.9%)	8 (57.1%)	
Male	29 (49.2%)	14 (50.0%)		8 (57.1%)	6 (42.9%)	
Age at diagnosis, Mean ± S.D.(year)	31.5 ± 2.279	30.1 ± 3.035	0.718	27.86 ± 4.436	32.29 ± 4.224	0.476
Lesion site			0.099			0.663
Craniofacial	27 (45.8%)	7 (25.0%)		4 (28.6%)	3 (21.4%)	
Extra-craniofacial	32 (54.2%)	21 (75.0%)		10 (71.4%)	11 (78.6%)	
Multiplicity			0.011			0.077
Monostotic	56 (94.9%)	21 (75.0%)		13 (92.9%)	8 (57.1%)	
Polyostotic	3 (5.1%)	7 (25.0%)		1 (7.1%)	6 (42.9%)	
Decalcification			0.001			0.326
No	12 (20.3%)	23 (82.1%)		10 (71.4%)	13 (92.9%)	
Yes	47 (79.7%)	5 (17.9%)		4 (28.6%)	1 (7.1%)

### *GNAS* mutation rate in fibrous dysplasia specimens with or without decalcification

The observed *GNAS* mutation rate in tissue samples from patients with FD (32.2%) was remarkably lower than that reported by a previous study (95%)^19^. Both studies used the same next-generation sequencing method, a pyrosequencing. Therefore, we hypothesized that mutation rates are affected by decalcification, and compared mutation rates according to whether or not the FD tissue samples were decalcified (Table 2). The observed *GNAS* mutation rate was much lower in FD tissue specimens that had been subjected to decalcification (5/52, 9.6%) than in FD specimens without decalcification (23/35, 65.7%, *P* = 0.001).

**Table 2:** Detection rate of *GNAS* mutation in tissue samples (with or without decalcification).

*GNAS* mutation	With decalcification	Without decalcification	*P*-value
**All fibrous dysplasia samples (n = 87)**			
Presence	5	23	
Absence	47	12	
Mutation rate	5/52 (9.6%)	23/35 (65.7%)	0.001
**Matched tissue samples (n = 15)**			
Presence	2	9	
Absence	13	6	
Mutation rate	2/15 (13.3%)	9/15 (60.0%)	0.021

To verify the effect of tissue decalcification on the detection rate of *GNAS* mutations by pyrosequencing, we divided tissue specimens from 15 FD patients into two parts: one part of each specimen was decalcified, and the other part was not decalcified. Subsequent tissue processing, PCR, and pyrosequencing reactions were the same for both tissue samples. Table 2 compares the observed *GNAS* mutation rates in these two tissues (decalcified or not) for the 15 FD patients. Consistent with the previous experiment, the observed *GNAS* mutation rates in decalcified tissue specimens were lower (2/15, 13.3%) that those in undercalcified tissue specimens (9/15, 60.0%, *P* = 0.021). These combined results indicate that *GNAS* mutation was not detected in 7 FD patients when the source of DNA for diagnostic pyrosequencing reactions was derived from decalcified tissue specimens.

### Clinical features of fibrous dysplasia according to *GNAS* mutation type

We detected *GNAS* mutation in 28 out of 87 FD lesions (Table 1). Fourteen of these patients had C > T substitution mutations (R201C) and the other 14 patients had G > A substitution mutations (R201H). Other rare mutations were not identified.

The R201H mutation occurred more frequently in polyostotic FD, whereas the R201C mutation occurred more frequently in monostotic FD (*P* = 0.077). There were no significant differences in gender, age at diagnosis, or lesion site between patients with R201H and R201C mutations in *GNAS* leading to FD.

## Discussion

*GNAS* encodes a subunit of the stimulatory cAMP pathway-associated G-protein, Gsα. Activating or gain-of-function *GNAS* mutations in osteoblastic cells result in adenylate cyclase activation and constitutive elevation of intracellular cyclic adenosine monophosphate (cAMP). Consequently, mutated osteoblastic cells undergo increased cell proliferation and reduced cell differentiation, resulting in overproduction of disorganized fibrotic bone matrix. This process is responsible for the development of FD, which may also present as a feature of McCune-Albright syndrome (endocrine diseases or Café au lait macules occurs in addition to FD)^22–25^.

The diagnosis of FD usually requires clinical information, radiographical images, and histological findings, but sometimes it can be challenging. In many cases, it can be difficult or impossible to distinguish FD from other fibro-osseous lesions only based on histological findings. Several studies reported that GNAS mutation was absent in other fibro-osseous disorders such as low-grade osteosarcoma, ossifying fibroma, and osteofibrous dysplasia, and therefore GNAS gene analysis can aid the differntiation of FD from such disease entities^15, 16, 26^.

A previous meta-analysis reported that the detection rate of *GNAS* mutation varied from 45–95%^15–19^, and the overall positive detection rate of *GNAS* mutation was 71.9% in 203 patients with FD^14^. In our study, pyrosequencing results detected *GNAS* mutations in only 32.2% of FD cases, regardless of decalcification. This result might be explained by differences in methodologies used to detect *GNAS* mutations, which result in different sensitivities. Several methods for detecting mutations have been published, including direct sequencing^7^, mutation-specific restriction enzyme digestion^13, 26^, PCR coupled with allele-specific oligonucleotide hybridization^6^, PCR–restriction fragment length polymorphism^27^, PCR with peptide nucleic acid^28^, and PCR with pyrosequencing^19^. A previous study performed pyrosequencing and reported the detection of *GNAS* mutations in 95% of FD patients, which is a much higher detection rate than that observed in the present study.

Next, we evaluated the effect of tissue decalcification on the detection of *GNAS* mutations in patients with FD. Many standard protocols for preparing bony tissue samples include treatment with strong acid to decalcify the tissue before fixation and embedding. This harsh treatment may affect the integrity of DNA isolated from the tissue, and thereby reduce the quantity and quality of DNA for PCR amplification and sequencing^21^. In this study, specimens were decalcified with Calci-Clear Rapid solution for 24 hours. The detection rate of *GNAS* mutations was 9.6% (5/52) in decalcified specimens and 65.7% (23/35) in non-decalcified specimens. A comparison of the detection rates of *GNAS* mutations in FD samples from 15 FD patients that were either decalcified or undercalcified also indicated that the detection rate of *GNAS* mutation was much higher in undercalcified tissues (9/15, 60.0%) than in decalcified tissues (2/15, 13.3%, *p* = 0.021).

The present study showed that patients with polyostotic FD more frequently displayed p.R201H mutation in *GNAS* than patients with monostotic FD (*p* = 0.077). This result was not consistent with a previous report that patients with polyostotic FD had R201C mutation in *GNAS* ^29^. We identified p.R201C mutation in one of seven patients with polyostotic FD. The lack of statistical significance in our result may be due to the small number of cases in our study.

In this study, we demonstrated that *GNAS* mutations were more frequently detected in FD tissue samples without decalcification than in decalcified FD tissue samples. And, most of the polyostotic FD patients displayed R201H substitution mutation in *GNAS*. Therefore, GNAS mutation test should be conducted without performing decalcification. Also, if sequencing analysis detects an R201H mutation in GNAS, we suggest that the clinician should consider the possibility of polyostotic FD.

## Materials and Methods

### Patients and samples

We retrospectively recruited 93 patients who were diagnosed with FD at Severance Hospital, Yonsei University College of Medicine, from 2006 to 2016. A total of 107 tissue samples were collected. The specimens included 72 decalcified FFPE specimens and 35 non-decalcified FFPE specimens. The DNA isolated from six patients was not suitable for molecular studies; therefore, we analyzed the DNA of 87 patients with FD. Ten patients presented with multiple bone lesions on radiological studies when FD was initially diagnosed, suggesting polyostotic FD. Among the ten polyostotic patients, only two patients underwent multiple biopsies and/or curettages from different locations, whereas the other patients received only one biopsy or curettage from a representative lesion. In 15 cases, only part of the specimen was subjected to decalcification and the rest was not decalcified, which enabled a direct comparison of the effect of decalcification on the detection of mutations in a tissue specimen. For decalcification, the tissue samples were decalcified with Calci-Clear Rapid solution containing EDTA and hydrochloric acid (National Diagnostics, Georgia, USA) for 24 hours, fixed in 10% formalin, and then embedded in paraffin.

To select the most representative FFPE tissues for molecular studies, samples were mounted on slides, stained with hematoxylin and eosin (H&E), and reviewed by two pathologists (SJS and SKK). Medical records were reviewed for clinical features, and radiological and pathological findings.

All methods and experimental protocols using human tissue (FFPE tissue) were carried out in accordance with relevant guidelines and regulations approved by the Institutional Review Board of Severance Hospital, Yonsei University Health System (4-2016-0276). The informed consent was waved because the IRB decided that this retrospective study had a minimal risk to the patients (risk level I).

### DNA extraction and PCR amplification

Genomic DNA was extracted from 10 μm sections cut from FFPE tissue blocks using the Maxwell® CSC DNA FFPE extraction kit (Promega, Wisconsin, USA) and the Maxwell® CSC instrument. The genomic DNA was subjected to PCR amplification optimized for pyrosequencing analysis; reactions contained 9 μl (10 ng/μl) of DNA, 12.5 μl of 2 × PyroMark PCR master mix (QIAGEN, Hilden, Germany), 2.5 μl of 10x CoralLoad PCR loading buffer (QIAGEN), and 1 μl each of the forward and reverse primers, in a total reaction volume of 25 μl (Supplementary Table 2). The following program was used for PCR amplification: 95 °C for 15 minutes; followed by 45 cycles of 94 °C for 30 seconds, 59 °C for 30 seconds, and 72 °C for 30 seconds; and a final extension step of 72 °C for 10 minutes.

### Pyrosequencing

After PCR analysis, single-stranded DNA suitable for pyrosequencing was prepared using the PyroMark Q24 MDx Vacuum Workstation (QIAGEN, Hilden, Germany). In workstation, the biotinylated PCR products were bound to streptavidin-coated sepharose beads. The beads were released into a PyroMark Q24 Plate containing 25 μl of annealing buffer and 0.3 μM of sequencing primer. The sample was heated to 80 °C for 2 minutes, and then cooled to room temperature. Pyrosequencing was performed in a PyroMark Q24 MDx (QIAGEN) according to the manufacturer’s instructions. Sequencing data were analyzed using PyroMark Q24 MDx Software 2.0 (QIAGEN).

### Statistical analysis

All statistical analyses were performed using SPSS version 18.0 (IBM, Chicago, IL, USA). The relationships between groups were compared using the χ^2^ test, Fisher’s exact test, or the Mann-Whitney U-test. Two-sided *P* values < 0.05 were considered as statistically significant.

## Electronic supplementary material

Supplementary Tables
