# Supplementary material for: Frequency of GNAS R201H substitution mutation in polyostotic fibrous dysplasia: Pyrosequencing analysis in tissue samples with or without decalcification
Source: Sci Rep. 2017 Jun 6;7:2836. doi: 10.1038/s41598-017-03093-1 (PMC5460223; doi:10.1038/s41598-017-03093-1)
Supplement: Supplementary file 1 — Supplementary Tables [file 41598_2017_3093_MOESM1_ESM.doc]

**Frequency of *GNAS* R201H substitution mutation in polyostotic fibrous dysplasia: Pyrosequencing analysis in tissue samples with or without decalcification**

Su-Jin Shin,^1^ Seok Joo Lee,^2^ & Sang Kyum Kim^2,*^

^1^Department of Pathology, Hanyang University College of Medicine, Seoul, Korea ^2^Department of Pathology, Yonsei University College of Medicine, Seoul, Korea

**Running title:** *GNAS* mutation in fibrous dysplasia

***Corresponding author**

Sang Kyum Kim, M.D., Ph.D.

Department of Pathology, Yonsei University College of Medicine

Yonsei-ro 50-1, Seodaemun-gu, Seoul, 03722, Korea

E-mail: NICEKYUMI@yuhs.ac

Tel: +82-2-8923-7845

Fax: +82-2-362-0860

**Supplementary Table 1.** Clinical features of 87 patients with fibrous dysplasia.

|  |  | *N* (%) |
| --- | --- | --- |
| Gender |  |  |
|  | Female | 44 (50.6) |
|  | Male | 43 (49.4) |
| Age at diagnosis, mean±S.D. |  | 31.02 ± 16.97 |
| Lesion site |  |  |
| Craniofacial |  | 34 (39.1) |
|  | Skull | 21 (24.1) |
|  | Maxilla | 7 (8.1) |
|  | Sphenoid sinus | 3 (3. 5) |
|  | Ethmoid sinus | 1 (1.2) |
|  | Mandible | 1 (1.2) |
|  | Orbit | 1 (1.2) |
| Extracraniofacial |  | 53 (60.9) |
|  | Femur | 21 (24.1) |
|  | Rib | 9 (10.3) |
|  | Humerus | 5 (5.8) |
|  | Pelvis | 5 (5.8) |
|  | Tibia | 4 (4.6) |
|  | Spine | 3 (3. 5) |
|  | Phalange (finger) | 3 (3.5) |
|  | Radius | 2 (2.3) |
|  | Ulna | 1 (1.2) |
| Multiplicity |  |  |
|  | Monostotic | 77 (88.5) |
|  | Polyostotic | 10 (11.5) |
| Decalcification |  |  |
|  | No | 35 (40.2) |
|  | Yes | 52 (59.8) |

**Supplementary Table 2.** Primers used for PCR amplification and sequencing

| Name | Primer sequence |
| --- | --- |
| Codon 201 |  |
| Forward | 5’ - GTTTCGGTTGGCTTTGGTGA - 3’ |
| Reverse (biotin) | Biotin - 5’ - CCTGGAACTTGGTCTCAAAGATT - 3’ |
| Sequencing | 5’ – TCAGGACCTGCTTCGC – 3’ |
| Codon 227 |  |
| Forward | 5’ - TTTCGGTTGGCTTTGGTGAG - 3’ |
| Reverse (biotin) | Biotin - 5’ - CACTTGCGGCGTTCATCG- 3’ |
| Sequencing | 5’ – GCATGTTTGACGTGGGT – 3’ |
